# Accessory Genome Dynamics of Local and Global *Staphylococcus pseudintermedius* Populations

**DOI:** 10.3389/fmicb.2022.798175

**Published:** 2022-02-10

**Authors:** Spencer A. Bruce, Joshua T. Smith, Jennifer L. Mydosh, John Ball, David B. Needle, Robert Gibson, Cheryl P. Andam

**Affiliations:** ^1^Department of Biological Sciences, University at Albany, State University of New York, Albany, NY, United States; ^2^Department of Molecular, Cellular and Biomedical Sciences, University of New Hampshire, Durham, NH, United States; ^3^Infectious Disease and Microbiome Program, Broad Institute of MIT and Harvard, Cambridge, MA, United States; ^4^New Hampshire Veterinary Diagnostic Laboratory, Durham, NH, United States

**Keywords:** *Staphylococcus pseudintermedius*, genome, methicilin resistance, multidrug resisitance, canine

## Abstract

*Staphylococcus pseudintermedius* is a major bacterial colonizer and opportunistic pathogen in dogs. Methicillin-resistant *S. pseudintermedius* (MRSP) continues to emerge as a significant challenge to maintaining canine health. We sought to determine the phylogenetic relationships of *S. pseudintermedius* across five states in the New England region of the United States and place them in a global context. The New England dataset consisted of 125 previously published *S. pseudintermedius* genomes supplemented with 45 newly sequenced isolates. The core genome phylogenetic tree revealed many deep branching lineages consisting of 142 multi-locus sequence types (STs). *In silico* detection of the *mecA* gene revealed 40 MRSP and 130 methicillin-susceptible *S. pseudintermedius* (MSSP) isolates. MRSP were derived from five structural types of SCC*mec*, the mobile genetic element that carries the *mecA* gene conferring methicillin resistance. Although many genomes were MSSP, they nevertheless harbored genes conferring resistance to many other antibiotic classes, including aminoglycosides, macrolides, tetracyclines and penams. We compared the New England genomes to 297 previously published genomes sampled from five other states in the United States and 13 other countries. Despite the prevalence of the clonally expanding ST71 found worldwide and in other parts of the United States, we did not detect it in New England. We next sought to interrogate the combined New England and global datasets for the presence of coincident gene pairs linked to antibiotic resistance. Analysis revealed a large co-circulating accessory gene cluster, which included *mecA* as well as eight other resistance genes [*aac (6′)-Ie-aph (2″)-Ia, aad (6), aph (3′)-IIIa, sat4, ermB, cat, blaZ*, and *tetM*]. Furthermore, MRSP isolates carried significantly more accessory genes than their MSSP counterparts. Our results provide important insights to the evolution and geographic spread of high-risk clones that can threaten the health of our canine companions.

## Introduction

*Staphylococcus pseudintermedius* is a major component of the normal cutaneous microflora in healthy domesticated dogs and is a frequent colonizer of the skin and mucosae (perineum and oral cavity) (Bannoehr and Guardabassi, 2012; Garbacz et al., 2013). It is also an opportunistic pathogen and is often implicated in skin and ear infections, urinary tract infections and post-surgical wounds (Bannoehr and Guardabassi, 2012; Garbacz et al., 2013). It does not typically colonize humans and other animals, but transmission from dogs to humans and other animal hosts (e.g., cats, horses, parrots) has been reported (Devriese et al., 2005; Stegmann et al., 2010; Laarhoven et al., 2011; van Duijkeren et al., 2011). Close contact between pet owners and veterinarians with infected dogs can facilitate cross-species transmission (Laarhoven et al., 2011; van Duijkeren et al., 2011; Somayaji et al., 2016). However, in a rare cluster of *S. pseudintermedius* infections documented in four elderly patients at a tertiary hospital with probable direct or indirect patient-to-patient transmission, no animal source was identified as the cause (Starlander et al., 2014). The bullous skin lesions found on two of the patients indicated the production of exfoliative toxin, a troublesome sign of its potential as an emerging zoonotic pathogen in humans (Starlander et al., 2014).

Similar to methicillin-resistant *Staphylococcus aureus* (MRSA) which is the leading cause of skin and soft tissue infections, endocarditis, bloodstream infections, pneumonia and bone and joint infections in humans (Turner et al., 2019), methicillin-resistant *S. pseudintermedius* (MRSP) continues to emerge as a significant challenge to supporting canine health (Lynch and Helbig, 2021). MRSP is often implicated in canine pyoderma and otitis externa (Lynch and Helbig, 2021). In dogs diagnosed with clinical infections, rates of MRSP can range from 7 to 38% (Couto et al., 2016; Nisa et al., 2019), and even an astounding 74% in dogs with superficial pyoderma (González-Domínguez et al., 2020), which can complicate treatment options (Bannoehr and Guardabassi, 2012; Garbacz et al., 2013). More problematic is that dogs can carry MRSP for more than a year after a clinically apparent infection, with systemic antibiotic treatment prolonging the duration of MRSP carriage (Windahl et al., 2012). The emergence of multidrug-resistant (MDR) MRSP strains further poses an important challenge to therapy in veterinary medicine (Lynch and Helbig, 2021).

The health and welfare of dogs and other companion animals, and the close interactions humans have with them, necessitates a deeper understanding of the bacterial pathogens that they harbor. Elucidating the origins, evolutionary history, and genetic basis of bacterial resistance from a genomic context is needed to explore patterns of dissemination at both local and global geographical scales. Moreover, a systematic effort to implement whole genome sequencing in veterinary medicine will be instrumental in advancing the One Health concept, centered on the interconnectedness of animal, human and environmental health (Zinsstag et al., 2011). Recently, we reported that the population structure of *S. pseudintermedius* in New England located in the northeastern part of the United States has been shaped by frequent recombination and long-distance dissemination (Smith et al., 2020). In the current study, we expanded the New England dataset to include additional newly sequenced genomes. We sought to determine the phylogenetic relationships of MRSP and MDR *S. pseudintermedius* across five states in New England and place them in a global context. We used 171 genomes from New England combined with 297 genomes from other parts of the United States and worldwide. We then used this combined dataset to identify co-circulating gene clusters related to MRSP and MDR in *S. pseudintermedius.* Our results suggest that the clonal expansion and geographical dissemination of certain sequence type (ST) were associated with the acquisition of resistance genes, which are part of a larger interconnected coincident accessory genome. Our results provide important insights into the evolution of high-risk clones and the geographical dissemination of resistance genes that can threaten the health of our canine companions.

## Materials and Methods

### Sample Collection in New England

The New England *S. pseudintermedius* collection consisted of 129 isolates that were previously published by our group and were sampled from October 2017 to October 2018 (Smith et al., 2020) as well as 69 new isolates sampled from July 2018 to June 2019. Methods for sample collection have been described previously (Smith et al., 2020). Briefly, isolates were obtained as culture swabs from routine clinical specimens submitted to the New Hampshire Veterinary Diagnostic Laboratory (NHVDL), New Hampshire, United States. The clinical specimens were received from different veterinary practices from the states of Connecticut, New Hampshire, Maine, Massachusetts, and Vermont, located in the northeastern part of the United States. All isolates were from animals diagnosed with clinical infections. As part of routine clinical submissions, the NHVDL was exempt from the IACUC approval process. We used commercially prepared tryptic soy agar with 10% sheep red blood cells to culture pure isolates. Initial species identification was carried out using matrix-assisted laser desorption/ionization time-of-flight mass spectrometry using the Bruker Biotyper instrument (Bruker Daltonics, Bremen, Germany) and following manufacturer’s protocols. Species assignments were made by comparing mass spectra of our samples to two libraries of reference spectra RUO library 7311 (V7) and 7854 (V8) available in the Bruker MBT Compass. The most common sites of bacterial isolation were skin, ears, urine and wounds. All isolates were stored in Brain Heart Infusion broth with DMSO at −80^°^C. Associated metadata information for each isolate is included in Supplementary Table 1.

### Cefoxitin and Oxacillin Susceptibility Screening

*In vitro* phenotypic screening for cefoxitin and oxacillin resistance was carried out using the Kirby Bauer disc diffusion technique. We followed the breakpoint guidelines of the most current Clinical and Laboratory Standards Institute for oxacillin and cefoxitin, which are used as the official predictors of methicillin resistance for *Staphylococcus* (Sweeney, 2018). For isolates identified as methicillin resistant, we also tested for the presence of the altered penicillin-binding protein PBP2a using a commercial latex agglutination test (MASTALEX MRSA Latex Kit, MAST, United Kingdom) following manufacturer’s protocols. Verification for the presence of the *mecA* gene, which encodes methicillin resistance, was done using whole genome sequencing (described below).

### DNA Extraction and Whole Genome Sequencing

DNA extraction was carried out using the Zymo Research Quick-DNA Fungal/Bacterial Miniprep Kit (Irvine, California) following the manufacturer’s protocol. We quantified DNA concentration using a Qubit fluorometer (Invitrogen, Grand Island, NY). We prepared DNA libraries using the Nextera XT protocol with 1 ng of genomic DNA per isolate. Samples were sequenced using the Illumina HiSeq platform (San Diego, California) to produce 250 bp paired end reads. Sequencing was carried out at the Hubbard Center for Genome Studies at the University of New Hampshire.

### Genome Assembly and Annotation

We assembled all genomes using Shovill v1.1.0.^1^ Shovill is a series of methods that includes subsampling read depth down to 150X, trimming adapters, correcting sequencing errors and assembling using SPAdes v3.13.0 (Bankevich et al., 2012). Next, we used QUAST v5.0.2 (Gurevich et al., 2013) and CheckM v1.1.3 (Parks et al., 2015) to assess the quality of our sequences and exclude genomes with < 90% completeness and > 5% contamination. We also excluded assemblies with > 200 contigs and an N50 < 40,000 bp from downstream analyses. Using these assembly thresholds, only 126 out of 130 genomes from reference (Smith et al., 2020) and 45 out of 69 newly sequenced genomes met the assembly thresholds we set. In total, our final New England dataset included 171 genomes. Genomes were annotated using Prokka v1.14.6 (Seemann, 2014). Finally, all sequences were compared to the *S. pseudintermedius* reference genome [National Center for Biotechnology Information (NCBI) Accession: NC_014925.1] using FastANI v1.32 to confirm species designation using the ≥ 95% in average nucleotide identity (ANI) threshold (Jain et al., 2018).

### Pan-Genome Analysis and Phylogenetic Reconstruction

We used Panaroo v1.2.7 (Tonkin-Hill et al., 2020) to characterize the pan-genome, consisting of core genes and accessory genes (Medini et al., 2005). To balance the tradeoff between inferring robust phylogenetic relationships vs. accounting for assembly errors, we only included core genes if they were present in ≥ 99% of the genomes. Nucleotide sequences of each orthologous gene family were aligned using Clustal Omega v1.2.4 (Sievers and Higgins, 2014) and concatenated to generate a core genome alignment. Phylogenetically informative single nucleotide polymorphisms (SNPs) in the core genome alignment were extracted using SNP-sites (Page et al., 2016). We used the core SNP alignment to construct a maximum likelihood phylogenetic tree using RAxML v8.2.12 (Stamatakis, 2014) employing a general time-reversible nucleotide substitution model (Tavaré, 1986) and four gamma categories for rate heterogeneity.

### *In silico* Sequence Typing, Detection of Resistance Genes and SCC*mec*

Using the contig files, we determined the multilocus ST for all genomes used in this study using the program mlst v2.19.0.^2^ STs pertain to allelic profiles that characterize nucleotide differences in partial sequences of seven single-copy housekeeping genes (Maiden et al., 1998). In *S. pseudintermedius*, these allelic profiles are based on the genes *tuf, cpn60, pta, purA, fdh, ack*, and *sar* (Solyman et al., 2013). Allelic profiles of the genomes used in this study were compared to those in the *S. pseudintermedius* MLST database.^3^ Allelic profiles that did not appear in the pubMLST database were subsequently submitted for curation and ST designation. We next screened for the presence of horizontally acquired antibiotic resistance genes using ABRicate v1.0.1^4^ and the Comprehensive Antibiotic Resistance Database (CARD) (Jia et al., 2016), utilizing an 80% nucleotide identity threshold. Finally, we used staphopia-sccmec v0.1^5^ to determine the presence, type and subtype of the mobile genetic element SCC*mec* (Petit and Read, 2018). Staphopia-sccmec adheres to the naming convention developed by the International Working Group on the Classification of Staphylococcal Cassette Chromosome Elements (IWG-SCC) (IWG-SCC, 2009). It aligns experimentally tested SCC*mec* typing primers against assembled genomes using BLAST + (v2.7.1+) (Altschul et al., 1990). Samples with a perfect match to primer pairs for a given amplicon were assigned an SCC*mec* type and subtype following the Kondo et al. (2007) algorithm that uses multiplex PCRs to identify the individual SCC*mec* components. Isolates that harbored the *mecA* gene but did not produce a SCC*mec* designation using the staphopia-sccmec typing scheme were additionally screened against the *ccr* genes from previously described SCC*mec* elements unique to *S. pseudintermedius* (Worthing et al., 2018a). Sequences of the *ccr* gene were extracted and queried against each genome using a > 85% sequence similarity threshold with BLASTN v2.10.1 (Altschul et al., 1990).

### Global Isolates of *Staphylococcus pseudintermedius*

To place the diversity and population structure of *S. pseudintermedius* in New England in a global context, we combined our genomes with previously published genomes from other parts of the United States and around the world. We downloaded and assembled 60 read pairs from the SRA archive as well as 130 high quality genome assemblies from NCBI in October 2020. We also downloaded 107 genome sequences from the PATRIC database in October 2020. Raw read pairs were assembled with Shovill using the same parameters described above, and all genomes were subjected to the same quality standards as those collected in New England. In all, the non-New England dataset comprised 297 genomes from five states in the United States (Texas, Washington, Tennessee, Wisconsin, and Georgia) and 13 other countries. Accession numbers and associated metadata of the 297 non-New England genomes are listed in Supplementary Table 2.

### Identification of Coincident Resistance and Accessory Genes

To determine if antibiotic resistance genes are co-circulating with each other accessory genes and each other, we used the program Coinfinder v1.0.7 (Whelan et al., 2020). Coinfinder detects genes which associate or dissociate with other genes using a Bonferroni-corrected Binomial exact test statistic of the expected and observed rates of gene-gene association. We ran Coinfinder twice, first on our combined dataset to identify all coincident associated gene pairs, and then a second time using the query flag to look specifically at coincident associated gene pairs involving *mecA.* Network diagrams of these coincident gene-to-gene relationship were visualized with Gephi v0.9.2 (Bastian et al., 2009). We then compared the number of accessory genes between MRSP and methicillin-susceptible *S. pseudintermedius* (MSSP) genomes to determine if they were statistically different using Welch’s *t*-test with R package ggstatsplot v0.8.0 (Patil and Powell, 2018). We also used a pan-genome-wide association study (pan-GWAS) approach to identify genes that are enriched in MRSP compared to MSSP isolates using the program Scoary v1.6.16 (Brynildsrud et al., 2016). Lastly, we used the program islandpath v1.0.0 to predict genomic islands in our bacterial genomes based on the presence of dinucleotide biases and mobility genes (Bertelli and Brinkman, 2018).

We used the default parameters for each program unless indicated otherwise.

## Results

### Phylogenetic Diversity of *Staphylococcus pseudintermedius* in New England

**FIGURE 1:**
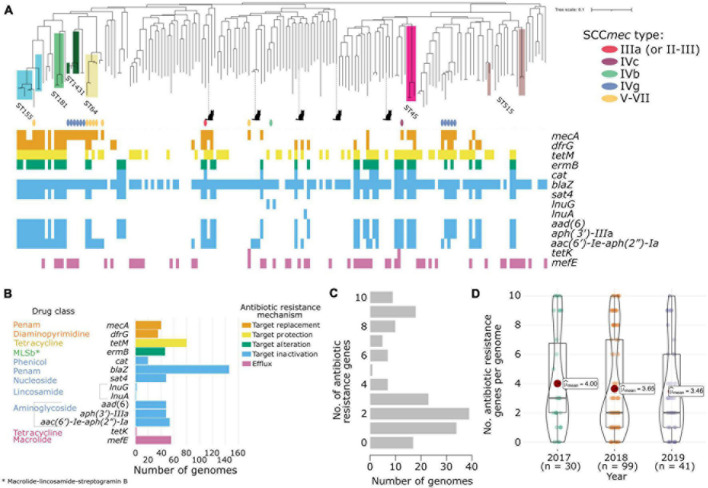
Antibiotic resistance characteristics of the 170 New England *S. pseudintermedius* population. **(A)** Gene presence–absence matrix showing the distribution of antimicrobial resistance genes present in each genome (colored blocks—present; white—absent). The midpoint-rooted maximum likelihood tree was built using sequence variation in 2,013 core genes. Scale bar represents the number of nucleotide substitutions per site. Also shown are the SCC*mec* types identified using staphopia-sccmec. For visual clarity, only STs represented by 3 or more genomes were labeled. ST information of other genomes in the tree are listed in Supplementary Table 1. Isolates from cats were depicted with a cat icon, while the rest came from dogs. **(B)** Bar plots showing the number of genomes that carry resistance genes in each drug class. The bars and gene names are colored according to the resistance mechanism defined by the CARD database. **(C)** Distribution of the number of acquired resistance genes per genome **(D)** Violin plots showing the number of resistance genes per genome per year of sampling. Associated box plots depict the minimum, first quartile, median, third quartile and maximum values, with data points shown.

We obtained a total of 170 high quality genomes of *S. pseudintermedius* isolates obtained through routine diagnostic tests of clinical specimens submitted to the NHVDL from October 2017 to June 2019 (Figure 1A and Supplementary Table 1). Of these, 125 came from a previously published study (Smith et al., 2020) and 45 that we have sequenced in this study. Isolates came from dogs (*n* = 165 isolates) and cats (*n* = 5 isolates). We obtained isolates from five states in New England: Connecticut (*n* = 2), New Hampshire (*n* = 112), Maine (*n* = 18), Massachusetts (*n* = 21), and Vermont (*n* = 18).

*De novo* assembly of the 170 genomes generated sequences of sizes ranging from 2.44 to 2.91 Mb (mean = 2.59 Mb). The number of predicted genes ranged from 2,231 to 2,797 (mean = 2,396) per genome (Supplementary Table 3). The pan-genome of the New England *S. pseudintermedius* population consisted of 4,553 orthologous gene families. These can be classified into core genes (*n* = 2,013 genes; present in 169–170 genomes), soft core genes (*n* = 49 genes; present in 162–168 genomes), shell genes (*n* = 641 genes; present in 26–161 genomes) and cloud genes (*n* = 1,850; present in 1–25 genomes). The combined core and soft-core genes comprised 45.29% of the pan-genome, while the combined shell and cloud genes (which together make up the accessory genome) comprised 54.71% of the pan-genome. We identified 264 genes unique to a single strain (or singletons) representing 5.80% of the pan-genome. We also sought to determine the degree of overall genomic relatedness among the *S. pseudintermedius* isolates. We calculated the ANI of all orthologous genes shared between any two genomes. Genome-wide ANI values for every possible pair of *S. pseudintermedius* genomes range from 98.84 to 100% (mean = 99.27%) (Supplementary Table 4). These results further reveal the large phylogenomic diversity present in the New England population.

The maximum likelihood phylogenetic tree based on the alignment of 72,152 SNPs of the core genes reveal many deep branching lineages consisting of 110 previously identified STs and 34 novel STs (Supplementary Table 1). The ST with the greatest number of *isolates* was ST155 (*n* = 7), while the rest consisted of less than four representatives. We detected 11 STs that were represented by two isolates and 125 STs that were represented by a single isolate. The five isolates from cats were found in disparate parts of the tree.

### Antibiotic Resistant Genes

*In silico* detection of the *mecA* gene from the genome sequences revealed 41 MRSP isolates and 130 MSSP isolates. The *mecA* gene encodes an extra penicillin-binding protein (PBP2a) that has low affinity to virtually all beta-lactam antibiotics (Katayama et al., 2000; IWG-SCC, 2009). We found some discrepancies between the *in vitro* phenotypic testing for methicillin resistance and the *in silico* detection of the *mecA* gene. There were five isolates whose genomes contained the *mecA* gene but were phenotypically tested as methicillin susceptible. There were 17 isolates whose genomes did not contain the *mecA* gene but were phenotypically tested as MRSP. Pan-GWAS analyses using Scoary v1.6.16 (Brynildsrud et al., 2016) failed to identify genes or mutations specific to methicillin resistance. None of the genes examined contained any SNPs across either group that harbored *mecA* but tested methicillin-susceptible or lacked *mecA* but tested methicillin-resistant. For comparison, a recent work on 592 isolates of *S. pseudintermedius* showed that *in silico* resistance typing corresponds to *in vitro* typing 98.4% of the time across 13 antibiotics in nine classes (Tyson et al., 2021). Moreover, the authors of that study also demonstrated that the correlation between *in vitro* and *in silico* typing for the *mecA* gene was slightly lower at 97.0%, and 12 of the 592 isolates (2.03%) examined were resistant to oxacillin without *mecA* present (Tyson et al., 2021). In contrast, our study produced similar but higher number of isolates (17 out of 170 or 10%) whose genomes did not contain the *mecA* gene phenotypically tested as MRSP. A similar finding has also been reported in four *S. aureus* isolates from the Scottish MRSA Reference Laboratory and which has been posited to indicate the existence of alternative mechanisms of beta-lactam resistance (e.g., due to amino acid substitutions of endogenous PBPs) (Ba et al., 2014). In our analysis, we found that out of the 171 genomes examined, only four isolates that were phenotypically tested as MRSP and harbored the *mec*A gene also carried the *mecA* regulator genes *mecI* and *mecR1*. None of the isolates examined harbored the *mecB* or *mecC* gene, both of which were reported to be associated with methicillin resistance or regulation in *S. aureus* (Lee et al., 2018). We also identified multiple instances where isolates tested as MRSP in both *in vitro* and *in silico* settings yet lacked an SCC*mec* designation (Supplementary Table 1). These results suggest the presence of other yet unknown genetic determinants or alternative mechanisms of methicillin resistance in databases. Other possible reasons to explain the discrepancies between phenotypic and genotypic results for methicillin resistance are sequencing errors, undocumented regulatory genes influencing expression, or the existence of novel *mecA* homologs not recognized as yet in current search engines.

The *mecA* gene is carried by a mobile chromosomal cassette SCC*mec* and are classified into types based on the combination of the *ccr* and *mec* complexes they carry (IWG-SCC, 2009). To date, 14 structurally variable SCC*mec* types (I–XIV) and several subtypes have been described (IWG-SCC, 2009; Lakhundi and Zhang, 2018; Urushibara et al., 2020). SCC*mec* typing of the New England *S. pseudintermedius* genomes revealed the presence of types IIIa (*n* = 1 genome), IVc (*n* = 1 genome), IVb (*n* = 1 genome), IVg (*n* = 11 genomes), V-VII (*n* = 7 genomes) (Figure 1A and Supplementary Table 1). These were distributed across different STs throughout the phylogeny. Because many of the known SCC*mec* types are based on those identified in *S. aureus* (IWG-SCC, 2009), our analysis is likely to underestimate the SCC*mec* diversity found in non-*aureus* species. Novel SCC*mec* elements have been identified in *S. pseudintermedius* that are not recorded in the current IWG-SCC database (Chanchaithong et al., 2016; Worthing et al., 2018a). We therefore used BLASTN to compare *ccr* gene sequences from previously identified *S. pseudintermedius* SCC*mec* elements against each genome assembly that harbored a *mecA* gene but lacked a designation from staphopia-sccmec and IWG-SCC. Results showed matches to three genomes (Supplementary Table 4). Two of these genomes (ST1602 and ST551) produced matches to *ccrA1* (90% sequence similarity) and *ccrB6* (100% sequence similarity) from a type designated as SCC*mec*_AI16_ (NCBI accession: LN864705) (Worthing et al., 2018a). The third genome (ST1492) produced a match to *ccrA1* (91% sequence similarity), but not to *ccrB6* for this same type. The observed sequence similarity to *ccr* genes previously reported as unique to *S. pseudintermedius* (Worthing et al., 2018b) highlights the need for further work on atypical SCC*mec* elements to understand the underlying mechanisms driving methicillin resistance in non-*aureus* species.

We determined the presence of other genes that confer resistance to different antibiotics as well. Based on the resistance mechanisms defined in the CARD database (Jia et al., 2016), we identified a total of 14 resistance genes present in both MRSP and MSSP genomes (MRSP determined by the presence of *mecA*) (Figures 1A,B and Supplementary Table 5). The most common resistance gene was *blaZ*, which is responsible for penicillin resistance. We detected *blaZ* in 146 genomes (or 85.88% of the population). The *tetM* gene, which confers tetracycline resistance, was detected in 79 genomes (46.47% of the population). Many MRSP and MSSP genomes also harbored genes conferring resistance to other antibiotic classes. Some resistance genes we detected were often associated with known STs. These included: trimethoprim resistance gene *dfrG* in 35 genomes (20.59%), macrolide resistance gene *mefE* found in 56 genomes (32.94%) and macrolide-lincosamide-streptogramin B (MLSb) resistance phenotype encoded by *ermB* detected in 46 genomes (27.06%). The plasmid-mediated streptothricin acetyltransferase gene *sat4*, aminoglycoside nucleotidyltransferase gene aad (6) and aminoglycoside phosphotransferase gene *aph* (3′)-IIIa were all detected in 48 genomes (28.24%). All five isolates from cats also carried resistance genes, ranging from 1 to 4 resistance genes per genome. A total of 39 genomes (or 22.94%) carried two antibiotic resistance genes. Of greatest concern is that 80 genomes harbored at least three resistance genes of which 11 had ten or more resistance genes (Figure 1C). Lastly, the number of resistance genes per genome was fairly consistent throughout the 3 years of sampling (Figure 1D). Only 17 genomes out of 170 do not carry any known transferrable resistance genes.

### Global Phylogenetic Relationships

**FIGURE 2:**
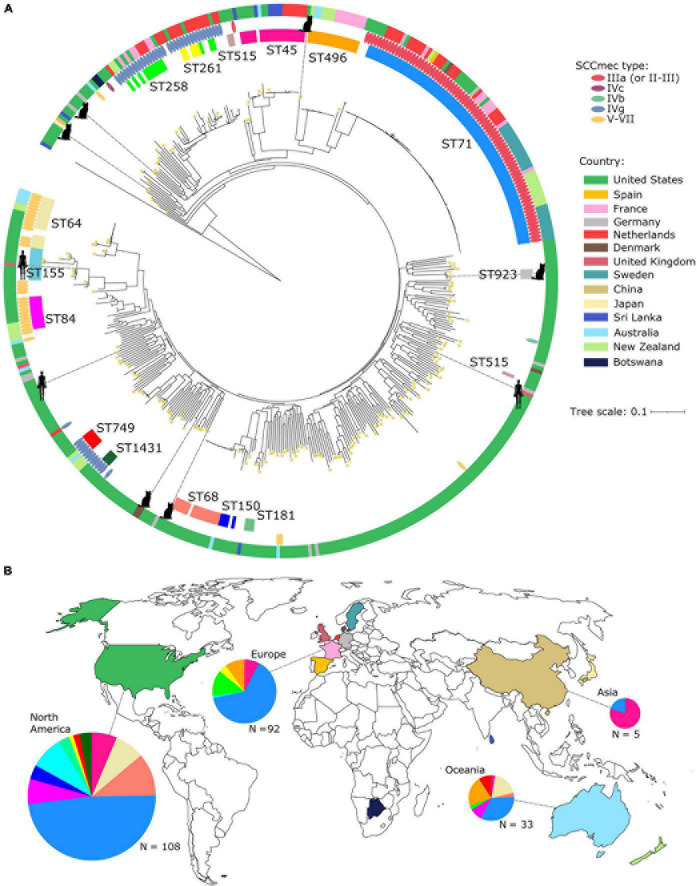
Core genome phylogenetic tree of the global *S. pseudintermedius* population. **(A)** Midpoint-rooted maximum likelihood tree showing the phylogenetic relationships of 468 *S. pseudintermedius* genomes. The tree was built using sequence variation in 1,634 core genes. Scale bar represents the number of nucleotide substitutions per site. Also shown are the STs, SCC*mec* types and country of origin. For visual clarity, only STs of MRSP from large phylogenetic clusters were labeled. Images of hosts were only shown for those isolates sampled from cats and humans, while the rest came from dogs. Yellow asterisks on branch tips represent genomes from New England. **(B)** Geographical distribution of STs. Colors of countries on the map correspond to the same colors in panel a. Colors in the pie charts correspond to the STs in panel a. Only proportions of the major STs that have SCC*mec* are shown in the pie charts. The size of the pie charts is proportional to the number of genomes.

We next sought to determine how the New England lineages were related to the global population of *S. pseudintermedius*. We combined 297 high quality genomes downloaded from NCBI and PATRIC databases to the 170 genomes from New England. In total, we included 467 genomes (Supplementary Table 2). Within the global dataset, a total of 296 came from the United States. We built a maximum likelihood phylogenetic tree using 212,612 SNPs from the sequence alignment of 2,005 core genes (Figure 2A). STs that made up large clusters in the tree included STs 45, 64, 68, 71, 84, 155, 258, 261, 496, and 749. We found that the New England MRSP lineages were widely distributed across the tree. There were MRSP STs that consisted of a mix of genomes from New England and other countries, as in the case of STs 45, 64, 155, 261, and 749, which were also found in the United Kingdom, Sri Lanka, Netherlands, Australia, and New Zealand.

Among the STs that make up the largest phylogenetic clusters in the tree, the most common was ST71 (*n* = 81 genomes representing 17.34% of the global dataset). In this dataset, all ST71 genomes originated from dogs. ST71 was present in three states in the United States (Tennessee, Washington, and Texas) and in seven other countries. Despite the prevalence of ST71 worldwide, it is curious that we did not detect it in New England (Figure 2B). ST71 appears to have had experienced a recent clonal expansion, which was reflected by the extremely short branches in the tree. Its clonal expansion may have been facilitated by the acquisition of SCC*mec* type IIIa (as defined by staphopia-sccmec and IWG-SCC database), and which was present in all ST71 genomes. This ST71-associated SCC*mec* type is often referred to as SCC*mec* type II-III hybrid (Descloux et al., 2008; Perreten et al., 2010), while other studies of ST71-associated SCC*mec* type II-III designate it simply as SCC*mec* III (Worthing et al., 2018a; Krapf et al., 2019). Such inconsistencies and difficulties in the nomenclature and classification of SCC*mec* elements in *S. pseudintermedius* highlight the need to establish a genus-wide SCC*mec* nomenclature system that encompasses more non-*aureus* species. This will ensure a standardized and systematic approach to precisely characterize novel and rare SCC*mec* variants in the future.

Other notable STs carried other SCC*mec* types and included ST 258, ST261, ST1431, and ST749 (*n* = 23; all carrying SCC*mec* type IVg). In addition, ST84 and ST64 all carried SCC*mec* type V–VII apart from a single ST64 genome. Results of the BLASTN analysis that we carried out to compare *ccr* genes from SCC*mec* types unique to *S. pseudintermedius* against the non-New England genomes resulted in matches across 20 genomes (Supplementary Table 6). Eighteen of these genomes (encompassing all of ST496, *n* = 16) and a single genome each from ST121 and ST930 produced matches to *ccrA1* (90% sequence similarity) and *ccrB6* (100% sequence similarity) from the aforementioned SCC*mec*_AI16_ type. A single genome from Texas (tamu_1470) produced significant matches to the *ccrB3* gene (94, 91, and 90% sequence similarity) from three previously described *S. pseudintermedius* SCC*mec* types SCC*mec*_AI16_, SCC*mec*_KM241_ (NCBI accession: AM904731) and SCC*mec*_KM1381_ (NCBI accession: AM904732), respectively (Descloux et al., 2008; Worthing et al., 2018a). One other genome (GCF_003383995.1_ASM338399v1) produced a significant match to the SCC*mec*_AI16_ *ccrB6* gene (99% sequence similarity) only.

While the majority of the genomes were of canine origin, the global dataset also included genomes derived from cats (*n* = 6, of which five are from New England) and humans (*n* = 3) (Figure 2A). These non-canine genomes intermingled with the genomes of canine *S. pseudintermedius* throughout the phylogeny. The human-derived *S. pseudintermedius* genomes were from distantly related STs (ST155, ST879 and ST1025; one from the United States and two from the United Kingdom). Of the six cat-derived *S. pseudintermedius* genomes, one came from Denmark and was of ST990. None of the non-canine hosts were associated with the more dominant ST71.

### Coincident Gene Clusters Associated With Methicillin-Resistant and Multidrug-Resistant *Staphylococcus pseudintermedius*

**FIGURE 3:**
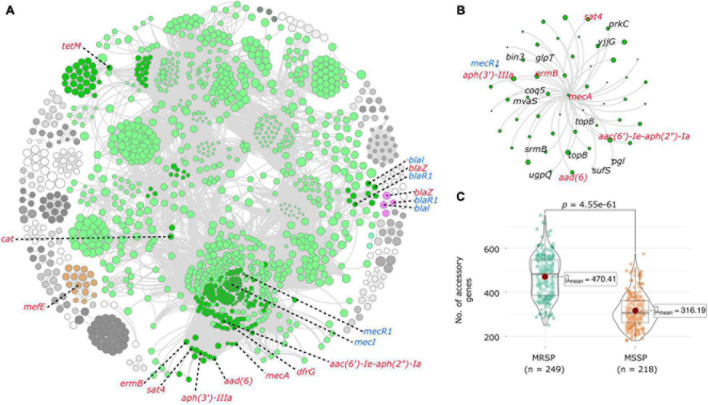
Coincident accessory and resistance genes. **(A)** Network diagram created with Gephi using output from Coinfinder carried out on 467 *S. pseudintermedius* genomes. Coincident gene clusters containing resistance genes are represented in color. The dark green nodes in the large green cluster represent genes associated with resistance genes. Resistance genes are labeled in red, and regulatory genes in blue. **(B)** Close-up view of the network that contains the *mecA* gene. Other resistance genes are colored in red, and genes with known functions are labeled in black. All other genes are hypothetical proteins. **(C)** Violin plots comparing the number of accessory genes in MRSP and MSSP isolates, created with ggstatplot. Violin plots depict the minimum, first quartile, median, third quartile and maximum values, with data points shown. The difference in the number of accessory genes is highly significant (*p* < 0.0001).

Coinfinder detected 47,784 statistically significant gene-to-gene relationships across the combined global dataset, resulting in 50 gene clusters. The largest gene cluster comprised 1,113 genes and included 10 antibiotic resistance genes (*mecA, tetM, cat, blaZ, aac (6′)-Ie-aph (2″)-Ia, dfrG, aad (6), APH (3′)-IIIa, sat4*, and *ermB*). A large number of genes from this cluster were hypothetical proteins (*n* = 926). A list of annotated genes (*n* = 187) from this cluster can be found in Supplementary Table 7. All other gene clusters were far smaller, ranging anywhere from 2 to 29 genes. Two other gene clusters also included antibiotic resistance genes, one made up of 16 genes including *mefE* and one made up of 4 genes including *blaZ*. Gene clusters that included *mecA* and *blaZ* also contained their known regulatory genes; *mecI*, *mecR1*, *blaI*, and *blaR1* (Figure 3A). When examining coincident gene-gene relationships involving *mecA* only, we identified 55 coincident genes, 18 known genes and 26 hypothetical proteins. Five of the genes directly coincident with *mecA* were antibiotic resistance genes; *aac (6′)-Ie-aph (2″)-Ia, aad (6), aph (3′)-IIIa, sat4, ermB* (Figure 3B). Three other resistance genes (*cat*, *blaZ*, and *tetM*) were part of the same larger coincident gene network but were not coincident with *mecA* directly. Other genes co-circulating directly with *mecA* were associated with a range of functions, including but not limited to the triggering of spore germination (*prkC*), DNA replication and transcription (*topB*), and RNA-stimulated ATP hydrolysis and RNA unwinding activity (*srmB*). A full list of coincident genes circulating directly with *mecA* and their annotations can be found in Supplementary Table 8. The statistical difference in number of accessory genes between MRSP and MSSP isolates as measured using Welch’s *t*-test was highly significant (*p* < 0.0001), with MRSP isolates harboring an average of more than 150 accessory genes compared to their MSSP counterparts (Figure 3C).

Results of the pan-GWAS analysis carried out to identify genes that are enriched in MRSP genomes largely reflected those of the coinfinder analysis with many of the same genes identified as significantly correlated. A full list of statistically significant genes associated with MRSP is found in Supplementary Table 9. The results of our pathogenicity island analysis resulted in the detection of anywhere from 0 to 13 genomic islands per isolate (mean = 1). The number of genomic islands seemed to be random when plotted in the context of the phylogenetic tree (Supplementary Figure 1), and there was not a significant trend between the number of antibiotic resistance genes and the number of pathogenicity islands predicted. A full list of isolates along with the coordinates of predicted genomic islands can be found in Supplementary Table 10.

## Discussion

We presented a population genomic analysis of clinical *S. pseudintermedius* isolates sampled from 2017 to 2019 across five states in the New England region of the United States and placed them in a global context. Our study revealed a genetically diverse set of isolates, both in terms of their genetic background and the antibiotic resistance genes they carry. Five structural types of SCC*mec* have contributed to the persistence of MRSP in the region, while *ccr* sequence similarity suggests potentially unique SCC*mec* elements that may be further influencing the MRSP population structure in New England and globally. In addition, we revealed a large number of co-circulating accessory genes associated with MRSP and MDR lineages, allowing for further studies into how coincident gene relationships may be affecting persistence, antibiotic resistance, and dissemination in this species.

MRSP has been spreading worldwide through the expansion and dissemination of specific lineages with different genetic backgrounds. Prior to 2010, population genetic studies of *S. pseudintermedius* reported that certain MRSP lineages predominate in different continents, as in the case of STs 71 and 258 in Europe, ST68 in North America, and STs 45 and 112 in Asia (Perreten et al., 2010; Pires dos Santos et al., 2016). However, the global population structure of MRSP has since been gradually changing and is becoming more heterogenous than previously recognized. In some parts of Europe, the apparent decline of ST71 appears to be concomitant with the emergence of two novel MRSP lineages of different origins: ST258 (from Northern Europe) and ST496 (from Australia) (Duim et al., 2016; Bergot et al., 2018). Moreover, ST71 now appears to have spread beyond the European borders to more distant locations. In New Zealand, the prevalence of ST71 in MRSP has been suggested to be a consequence of importation from other countries and autochthonous transmission within the country (Nisa et al., 2019). Importation of ST71 from other countries has also been postulated to explain its prevalence in Australia (Worthing et al., 2018b). The MRSP population in other parts of the world is more diverse. In Texas located in the southern part of the United States, the most prevalent MRSP STs were STs 64, 68, 71, and 84 (Little et al., 2019). Similarly, the MRSP population in Argentina consisted of genetically distinct STs not closely related to the more prevalent STs 71 and 68 (Gagetti et al., 2019). These included STs 339, 649, 919, 920, 921, and 922 that have not been reported elsewhere and were inferred to represent locally evolved clones (Gagetti et al., 2019). Our current study further corroborates these recent findings. Combined with our data, we postulate that the observed change in the global population structure *S. pseudintermedius* can be partly attributed to the increasing mobility of animals and people across geographical boundaries. The rapid spread of horizontally transferrable resistance genes is also a major contributing factor. Further dissemination of resistance genes can occur through subsequent transfers to other strains and/or vertical inheritance in descendant strains (McCarthy et al., 2015; Frosini et al., 2020). Thus, it is likely that it is only a matter of time before the globally dominant ST71 will be detected in New England, given its increased prevalence in the United States and abroad, as well as its unique adaptation in terms of increased adherence to canine corneocytes on the skin (Perreten et al., 2010; Latronico et al., 2014). It is clear that there is a need for enhanced *S. pseudintermedius* surveillance to rapidly identify and monitor the geographical spread of emerging MRSP and MDR lineages, including phenotypically tested MSSP that may carry other resistance genes.

The identification of coincident gene-to-gene relationships offers an important first step to understanding the role accessory genes play in the geographical dissemination and evolution of this opportunistic pathogen. Our results also demonstrate that portions of the accessory genome in MRSP isolates act as a pathogenicity island, although these genes may be more closely associated with virulence than antibiotic resistance given the lack of correlation between the two measures. By broadening our understanding of how accessory genes interact with one another, we can determine targeted approaches to treatment for MDR pathogens (Croll and McDonald, 2012). These findings offer a starting point for future researchers looking to build on the role co-circulating genes play in regulating, maintaining, and disseminating antibiotic resistance. Given that an accessory genome may be influenced by a wide variety of factors, a more detailed understanding of how certain genes are correlated can help us unravel their evolutionary origins (Jackson et al., 2011).

We acknowledge the imperfect nature of the New England dataset we used. The manner of the collection and sampling bias owing to the inclusion of clinical samples that have been sent to NHVDL by veterinary practices throughout the region meant that sampling may have missed some less common genotypes, certain regions in New England or other animal species. Our dataset was also biased toward those that were from animals diagnosed with infection, which meant we lacked information about *S. pseudintermedius* carriage in healthy animals. Because we only had less than 3 years of sampling, it is unclear whether the MRSP STs in New England represent long-standing variation or transient introductions. We also had disproportionately higher number of isolates from New Hampshire where NHVDL is located compared to the four other states in New England. This prevented statistical comparisons between individual states and thus should be considered when extrapolating information on gene prevalence compared to the broader *S. pseudintermedius* population. Similarly, the global dataset was biased toward certain countries and continents that report genomic data; hence, some geographical regions such as Africa and South America were poorly represented in our analyses. There were also fewer MSSP genomes relative to MRSP that were publicly available, which limited our ability to explore the phylogenetic and genomic differences between MRSP and MSSP. Another limitation of the study is the lack of information about the history of antibiotic use, treatment received or clinical outcomes of the hosts (animal or human) from which bacterial genomes were obtained. Hence, it is difficult to precisely deduce the contributions of specific *S. pseudintermedius* clones to disease outcomes. Nonetheless, our analyses revealed valuable insights and provided essential data to motivate the wider application of population genomics methods to support disease surveillance in veterinary medicine.

We expect that our study will foster new directions and opportunities to integrate whole genome sequencing approaches in veterinary medicine. Long-term, systematic genomic surveillance of *S. pseudintermedius* is needed to monitor the emergence and success of high-risk resistant clones. The widespread distribution of different MRSP lineages across continents is likely associated with cross-country importation of animals. While dogs are the natural hosts of *S. pseudintermedius*, the range of secondary hosts remains to be investigated. Future work should also focus on the association, if any, of the breed of dogs and *S. pseudintermedius* diversity, as well as bacterial differences between pets in homes, shelter dogs, stray dogs, working dogs (e.g., sheep dogs, rescue dogs, guide dogs, police dogs) and wild dogs. The genetic diversity of *S. pseudintermedius* in dogs may reflect the hygiene and social behavior patterns of the canine host, which will likely influence the frequency of exposure between dogs and other hosts (Bannoehr and Guardabassi, 2012). Such information will be critical to developing targeted strategies to control bacterial diseases in specific groups of dogs and in different settings. Whole genome sequencing will also be particularly important in clarifying cases of human infections where *S. pseudintermedius* had been previously misidentified as *S. aureus* (Börjesson et al., 2015). Lastly, understanding the genetic basis and drivers of host switching is critical to reducing the spread of resistance and to advancing the health of companion animals and the people who care for them.

In summary, our data show that *S. pseudintermedius* has diversified into multiple MRSP and MDR clones that have disseminated across New England and worldwide. This diversity is driven by the independent acquisitions of five structural types of the mobile SCC*mec* element, the spread of co-circulating antibiotic resistance gene clusters and the clonal expansion of certain MRSP clones. The diversity of *S. pseudintermedius* associated with canine infections and the existence of a wide array of transferable resistance genes warrants careful attention. Our study has important implications for both animal and human health, including epidemiological tracking, disease control and treatment of resistant bacteria.

## Data Availability Statement

The datasets presented in this study can be found in online repositories. The names of the repository/repositories and accession number(s) can be found in the article/Supplementary Material.

## Author Contributions

CA designed the work. SB carried out all bioinformatics analyses. JS, JM, JB, DBN, and RG carried out sampling, culturing, and DNA extractions. CA and SB wrote the manuscript with contributions from all authors. CA guided the work. All authors read, edited, and approved the final manuscript.

## Conflict of Interest

The authors declare that the research was conducted in the absence of any commercial or financial relationships that could be construed as a potential conflict of interest.

## Publisher’s Note

All claims expressed in this article are solely those of the authors and do not necessarily represent those of their affiliated organizations, or those of the publisher, the editors and the reviewers. Any product that may be evaluated in this article, or claim that may be made by its manufacturer, is not guaranteed or endorsed by the publisher.

## Footnotes

1
https://github.com/tseemann/shovill

2
https://github.com/tseemann/mlst

3
https://pubmlst.org/organisms/staphylococcus-pseudintermedius/

4
https://github.com/tseemann/abricate

5
https://github.com/staphopia/staphopia-sccmec

## References

[B1] Altschul S. F. Gish W. Miller W. Myers E. W. Lipman D. J. (1990). Basic local alignment search tool. *J. Mol. Biol.* 215 403–410. 2231712 10.1016/S0022-2836(05)80360-2

[B2] Ba X. Harrison E. M. Edwards G. F. Holden M. T. Larsen A. R. Petersen A. (2014). Novel mutations in penicillin-binding protein genes in clinical Staphylococcus aureus isolates that are methicillin resistant on susceptibility testing, but lack the mec gene. *J. Antimicrob. Chemother.* 69 594–597. 10.1093/jac/dkt418 24216768 PMC3922151

[B3] Bankevich A. Nurk S. Antipov D. Gurevich A. A. Dvorkin M. Kulikov A. S. (2012). SPAdes: a new genome assembly algorithm and its applications to single-cell sequencing. *J. Computat. Biol.* 19 455–477. 10.1089/cmb.2012.0021 22506599 PMC3342519

[B4] Bannoehr J. Guardabassi L. (2012). Staphylococcus pseudintermedius in the dog: taxonomy, diagnostics, ecology, epidemiology and pathogenicity. *Vet. Dermatol.* 23 253–e52. 10.1111/j.1365-3164.2012.01046.x 22515504

[B5] Bastian M. Heymann S. Jacomy M. (2009). “Gephi: an open source software for exploring and manipulating networks,” in *Third international AAAI conference on weblogs and social media*, (California, CA: AAAI).

[B6] Bergot M. Martins-Simoes P. Kilian H. Châtre P. Worthing K. A. Norris J. M. (2018). Evolution of the population structure of Staphylococcus pseudintermedius in France. *Front. Microbiol.* 9:3055. 10.3389/fmicb.2018.03055 30619143 PMC6300469

[B7] Bertelli C. Brinkman F. S. (2018). Improved genomic island predictions with IslandPath-DIMOB. *Bioinformatics* 34 2161–2167. 10.1093/bioinformatics/bty095 29905770 PMC6022643

[B8] Börjesson S. Gómez-Sanz E. Ekström K. Torres C. Grönlund U. (2015). Staphylococcus pseudintermedius can be misdiagnosed as Staphylococcus aureus in humans with dog bite wounds. *Eur. J. Clin. Microbiol. Infect. Dis.* 34 839–844. 10.1007/s10096-014-2300-y 25532507

[B9] Brynildsrud O. Bohlin J. Scheffer L. Eldholm V. (2016). Rapid scoring of genes in microbial pan-genome-wide association studies with Scoary. *Genome Biol.* 17 1–9. 27887642 10.1186/s13059-016-1108-8 PMC5124306

[B10] Chanchaithong P. Prapasarakul N. Perreten V. Schwendener S. (2016). Characterization of a novel composite staphylococcal cassette chromosome mec in methicillin-resistant Staphylococcus pseudintermedius from Thailand. *Antimicrob. Agents Chemother.* 60 1153–1157. 10.1128/AAC.02268-15 26643350 PMC4750692

[B11] Couto N. Monchique C. Belas A. Marques C. Gama L. T. Pomba C. (2016). Trends and molecular mechanisms of antimicrobial resistance in clinical staphylococci isolated from companion animals over a 16 year period. *J. Antimicrob. Chemother.* 71 1479–1487. 10.1093/jac/dkw029 26944924

[B12] Croll D. McDonald B. A. (2012). The accessory genome as a cradle for adaptive evolution in pathogens. *PLoS Pathog.* 8:e1002608. 10.1371/journal.ppat.1002608 22570606 PMC3343108

[B13] Descloux S. Rossano A. Perreten V. (2008). Characterization of new staphylococcal cassette chromosome mec (SCC mec) and topoisomerase genes in fluoroquinolone-and methicillin-resistant Staphylococcus pseudintermedius. *J. Clin. Microbiol.* 46 1818–1823. 10.1128/JCM.02255-07 18305127 PMC2395120

[B14] Devriese L. A. Vancanneyt M. Baele M. Vaneechoutte M. De Graef E. Snauwaert C. (2005). Staphylococcus pseudintermedius sp. nov., a coagulase-positive species from animals. *Int. J. Systemat. Evolut. Microbiol.* 55 1569–1573. 10.1099/ijs.0.63413-0 16014483

[B15] Duim B. Verstappen K. M. Broens E. M. Laarhoven L. M. Van Duijkeren E. Hordijk J. (2016). Changes in the population of methicillin-resistant Staphylococcus pseudintermedius and dissemination of antimicrobial-resistant phenotypes in the Netherlands. *J. Clin. Microbiol.* 54 283–288. 10.1128/JCM.01288-15 26582835 PMC4733195

[B16] Frosini S. M. Bond R. McCarthy A. J. Feudi C. Schwarz S. Lindsay J. A. (2020). Genes on the move: in vitro transduction of antimicrobial resistance genes between human and canine staphylococcal pathogens. *Microorganisms* 8:2031. 10.3390/microorganisms8122031 33353175 PMC7766859

[B17] Gagetti P. Wattam A. R. Giacoboni G. De Paulis A. Bertona E. Corso A. (2019). Identification and molecular epidemiology of methicillin resistant Staphylococcus pseudintermedius strains isolated from canine clinical samples in Argentina. *BMC Vet. Res.* 15 1–12. 10.1186/s12917-019-1990-x 31351494 PMC6660709

[B18] Garbacz K. Żarnowska S. Piechowicz L. Haras K. (2013). Staphylococci isolated from carriage sites and infected sites of dogs as a reservoir of multidrug resistance and methicillin resistance. *Curr. Microbiol.* 66 169–173. 10.1007/s00284-012-0254-9 23099429

[B19] González-Domínguez M. S. Carvajal H. D. Calle-Echeverri D. A. Chinchilla-Cárdenas D. (2020). Molecular Detection and Characterization of the mecA and nuc genes from staphylococcus species (S. aureus, S. pseudintermedius, and S. schleiferi) isolated from dogs suffering superficial pyoderma and their antimicrobial resistance profiles. *Front. Vet. Sci.* 7:376. 10.3389/fvets.2020.00376 32793641 PMC7390895

[B20] Gurevich A. Saveliev V. Vyahhi N. Tesler G. (2013). QUAST: quality assessment tool for genome assemblies. *Bioinformatics* 29 1072–1075. 10.1093/bioinformatics/btt086 23422339 PMC3624806

[B21] IWG-SCC (2009). Classification of staphylococcal cassette chromosome mec (SCCmec): guidelines for reporting novel SCCmec elements. *Antimicrob. Agents Chemother.* 53 4961–4967. 10.1128/AAC.00579-09 19721075 PMC2786320

[B22] Jackson R. W. Vinatzer B. Arnold D. L. Dorus S. Murillo J. (2011). The influence of the accessory genome on bacterial pathogen evolution. *Mobile Genet. Elements* 1 55–65. 10.4161/mge.1.1.16432 22016845 PMC3190274

[B23] Jain C. Rodriguez-R L. M. Phillippy A. M. Konstantinidis K. T. Aluru S. (2018). High throughput ANI analysis of 90K prokaryotic genomes reveals clear species boundaries. *Nat. Commun.* 9 1–8. 30504855 10.1038/s41467-018-07641-9 PMC6269478

[B24] Jia B. Raphenya A. R. Alcock B. Waglechner N. Guo P. Tsang K. K. (2016). CARD 2017: expansion and model-centric curation of the comprehensive antibiotic resistance database. *Nucleic Acids Res.* 2016:gkw1004. 10.1093/nar/gkw1004 27789705 PMC5210516

[B25] Katayama Y. Ito T. Hiramatsu K. (2000). A new class of genetic element, staphylococcus cassette chromosome mec, encodes methicillin resistance in Staphylococcus aureus. *Antimicrob. Agents Chemother.* 44 1549–1555. 10.1128/AAC.44.6.1549-1555.2000 10817707 PMC89911

[B26] Kondo Y. Ito T. Ma X. X. Watanabe S. Kreiswirth B. N. Etienne J. (2007). Combination of multiplex PCRs for staphylococcal cassette chromosome mec type assignment: rapid identification system for mec, ccr, and major differences in junkyard regions. *Antimicrob. Agents Chemother.* 51 264–274. 10.1128/AAC.00165-06 17043114 PMC1797693

[B27] Krapf M. Müller E. Reissig A. Slickers P. Braun S. D. Müller E. (2019). Molecular characterisation of methicillin-resistant Staphylococcus pseudintermedius from dogs and the description of their SCCmec elements. *Vet. Microbiol.* 233 196–203. 10.1016/j.vetmic.2019.04.002 31053353

[B28] Laarhoven L. M. De Heus P. Van Luijn J. Duim B. Wagenaar J. A. van Duijkeren E. (2011). Longitudinal study on methicillin-resistant Staphylococcus pseudintermedius in households. *PLoS One* 6:e27788. 10.1371/journal.pone.0027788 22132141 PMC3223215

[B29] Lakhundi S. Zhang K. (2018). Methicillin-resistant Staphylococcus aureus: molecular characterization, evolution, and epidemiology. *Clin. Microbiol. Rev.* 31 e20–e18. 10.1128/CMR.00020-18 30209034 PMC6148192

[B30] Latronico F. Moodley A. Nielsen S. S. Guardabassi L. (2014). Enhanced adherence of methicillin-resistant Staphylococcus pseudintermedius sequence type 71 to canine and human corneocytes. *Vet. Res.* 45 1–7. 10.1186/1297-9716-45-70 24957656 PMC4087241

[B31] Lee A. S. de Lencastre H. Garau J. Kluytmans J. Malhotra-Kumar S. Peschel A. (2018). Methicillin-resistant Staphylococcus aureus. *Nat. Rev. Dis. Primers* 4 1–23. 10.1385/1-59745-468-0:1 29849094

[B32] Little S. V. Bryan L. K. Hillhouse A. E. Cohen N. D. Lawhon S. D. (2019). Characterization of agr Groups of Staphylococcus pseudintermedius Isolates from Dogs in Texas. *Msphere* 4 e33–e19. 10.1128/mSphere.00033-19 30918056 PMC6437270

[B33] Lynch S. A. Helbig K. J. (2021). The Complex Diseases of Staphylococcus pseudintermedius in Canines: Where to Next? *Vet. Sci.* 8:11. 10.3390/vetsci8010011 33477504 PMC7831068

[B34] Maiden M. C. Bygraves J. A. Feil E. Morelli G. Russell J. E. Urwin R. (1998). Multilocus sequence typing: a portable approach to the identification of clones within populations of pathogenic microorganisms. *Proc. Natl. Acad. Sci.* 95 3140–3145. 10.1073/pnas.95.6.3140 9501229 PMC19708

[B35] McCarthy A. J. Harrison E. M. Stanczak-Mrozek K. Leggett B. Waller A. Holmes M. A. (2015). Genomic insights into the rapid emergence and evolution of MDR in Staphylococcus pseudintermedius. *J. Antimicrob. Chemother.* 70 997–1007. 10.1093/jac/dku496 25527273

[B36] Medini D. Donati C. Tettelin H. Masignani V. Rappuoli R. (2005). The microbial pan-genome. *Curr. Opin. Genet. Dev.* 15 589–594. 16185861 10.1016/j.gde.2005.09.006

[B37] Nisa S. Bercker C. Midwinter A. C. Bruce I. Graham C. F. Venter P. (2019). Combining MALDI-TOF and genomics in the study of methicillin resistant and multidrug resistant Staphylococcus pseudintermedius in New Zealand. *Sci. Rep.* 9 1–13. 10.1038/s41598-018-37503-9 30718644 PMC6361924

[B38] Page A. J. Taylor B. Delaney A. J. Soares J. Seemann T. Keane J. A. (2016). SNP-sites: rapid efficient extraction of SNPs from multi-FASTA alignments. *Microb. Genomics* 2:e000056. 10.1099/mgen.0.000056 28348851 PMC5320690

[B39] Parks D. H. Imelfort M. Skennerton C. T. Hugenholtz P. Tyson G. W. (2015). CheckM: assessing the quality of microbial genomes recovered from isolates, single cells, and metagenomes. *Genome Res.* 25 1043–1055. 10.1101/gr.186072.114 25977477 PMC4484387

[B40] Patil I. Powell C. (2018). *ggstatsplot:“ggplot2” based plots with statistical details.* Vienna: CRAN.

[B41] Perreten V. Kadlec K. Schwarz S. Grönlund Andersson U. Finn M. Greko C. (2010). Clonal spread of methicillin-resistant Staphylococcus pseudintermedius in Europe and North America: an international multicentre study. *J. Antimicrob. Chemother.* 65 1145–1154. 10.1093/jac/dkq078 20348087

[B42] Petit R. A. III Read T. D. (2018). Staphylococcus aureus viewed from the perspective of 40,000+ genomes. *PeerJ* 6:e5261. 10.7717/peerj.5261 30013858 PMC6046195

[B43] Pires dos Santos T. Damborg P. Moodley A. Guardabassi L. (2016). Systematic review on global epidemiology of methicillin-resistant Staphylococcus pseudintermedius: inference of population structure from multilocus sequence typing data. *Front. Microbiol.* 7:1599. 10.3389/fmicb.2016.01599 27803691 PMC5067483

[B44] Seemann T. (2014). Prokka: rapid prokaryotic genome annotation. *Bioinformatics* 30 2068–2069. 10.1093/bioinformatics/btu153 24642063

[B45] Sievers F. Higgins D. G. (2014). “Clustal Omega, accurate alignment of very large numbers of sequences,” in *Multiple sequence alignment methods*, (Totowa, NJ: Humana Press), 105–116. 10.1007/978-1-62703-646-7_6 24170397

[B46] Smith J. T. Amador S. McGonagle C. J. Needle D. Gibson R. Andam C. P. (2020). Population genomics of Staphylococcus pseudintermedius in companion animals in the United States. *Commun. Biol.* 3 1–11. 10.1038/s42003-020-1009-y 32503984 PMC7275049

[B47] Solyman S. M. Black C. C. Duim B. Perreten V. Van Duijkeren E. Wagenaar J. A. (2013). Multilocus sequence typing for characterization of Staphylococcus pseudintermedius. *J. Clin. Microbiol.* 51 306–310. 10.1128/JCM.02421-12 23115265 PMC3536184

[B48] Somayaji R. Priyantha M. A. R. Rubin J. E. Church D. (2016). Human infections due to Staphylococcus pseudintermedius, an emerging zoonosis of canine origin: report of 24 cases. *Diagnos. Microbiol. Infect. Dis.* 85 471–476. 10.1016/j.diagmicrobio.2016.05.008 27241371

[B49] Stamatakis A. (2014). RAxML version 8: a tool for phylogenetic analysis and post-analysis of large phylogenies. *Bioinformatics* 30 1312–1313. 10.1093/bioinformatics/btu033 24451623 PMC3998144

[B50] Starlander G. Börjesson S. Grönlund-Andersson U. Tellgren-Roth C. Melhus Å (2014). Cluster of infections caused by methicillin-resistant Staphylococcus pseudintermedius in humans in a tertiary hospital. *J. Clin. Microbiol.* 52 3118–3120. 10.1128/JCM.00703-14 24871217 PMC4136194

[B51] Stegmann R. Burnens A. Maranta C. A. Perreten V. (2010). Human infection associated with methicillin-resistant Staphylococcus pseudintermedius ST71. *J. Antimicrob. Chemother.* 65 2047–2048. 10.1093/jac/dkq241 20601356

[B52] Sweeney M. T. (2018). *CLSI performance standards for antimicrobial disk and dilution susceptibility tests for bacteria isolated from animals.* Wayne, PA: Clinical and Laboratory Standards Institute.

[B53] Tavaré S. (1986). Some probabilistic and statistical problems in the analysis of DNA sequences. *Lectures Math. Life Sci.* 17 57–86.

[B54] Tonkin-Hill G. MacAlasdair N. Ruis C. Weimann A. Horesh G. Lees J. A. (2020). Producing polished prokaryotic pangenomes with the Panaroo pipeline. *Genome Biol.* 21 1–21. 10.1186/s13059-020-02090-4 32698896 PMC7376924

[B55] Turner N. A. Sharma-Kuinkel B. K. Maskarinec S. A. Eichenberger E. M. Shah P. P. (2019). Methicillin-resistant Staphylococcus aureus: an overview of basic and clinical research. *Nat. Rev. Microbiol.* 17 203–218. 10.1038/s41579-018-0147-4 30737488 PMC6939889

[B56] Tyson G. H. Ceric O. Guag J. Nemser S. Borenstein S. Slavic D. (2021). Genomics accurately predicts antimicrobial resistance in Staphylococcus pseudintermedius collected as part of Vet-LIRN resistance monitoring. *Vet. Microbiol.* 254:109006. 10.1016/j.vetmic.2021.109006 33581494 PMC11555765

[B57] Urushibara N. Aung M. S. Kawaguchiya M. Kobayashi N. (2020). Novel staphylococcal cassette chromosome mec (SCC mec) type XIV (5A) and a truncated SCC mec element in SCC composite islands carrying speG in ST5 MRSA in Japan. *J. Antimicrob. Chemother.* 75 46–50. 10.1093/jac/dkz406 31617906

[B58] van Duijkeren E. Kamphuis M. Van der Mije I. C. Laarhoven L. M. Duim B. Wagenaar J. A. (2011). Transmission of methicillin-resistant Staphylococcus pseudintermedius between infected dogs and cats and contact pets, humans and the environment in households and veterinary clinics. *Vet. Microbiol.* 150 338–343. 10.1016/j.vetmic.2011.02.012 21420256

[B59] Whelan F. J. Rusilowicz M. McInerney J. O. (2020). Coinfinder: detecting significant associations and dissociations in pangenomes. *Microb. Genom.* 6:e000338. 10.1099/mgen.0.000338 32100706 PMC7200068

[B60] Windahl U. Reimegård E. Holst B. S. Egenvall A. Fernström L. Fredriksson M. (2012). Carriage of methicillin-resistant Staphylococcus pseudintermedius in dogs–a longitudinal study. *BMC Vet. Res.* 8 1–8. 10.1186/1746-6148-8-34 22444911 PMC3325892

[B61] Worthing K. A. Abraham S. Coombs G. W. Pang S. Saputra S. Jordan D. (2018a). Clonal diversity and geographic distribution of methicillin-resistant Staphylococcus pseudintermedius from Australian animals: discovery of novel sequence types. *Vet. Microbiol.* 213 58–65. 10.1016/j.vetmic.2017.11.018 29292005

[B62] Worthing K. A. Schwendener S. Perreten V. Saputra S. Coombs G. W. Pang S. (2018b). Characterization of staphylococcal cassette chromosome mec elements from methicillin-resistant Staphylococcus pseudintermedius infections in Australian animals. *Msphere* 3 e491–e418. 10.1128/mSphere.00491-18 30404937 PMC6222048

[B63] Zinsstag J. Schelling E. Waltner-Toews D. Tanner M. (2011). From “one medicine” to “one health” and systemic approaches to health and well-being. *Prevent. Vet. Med.* 101 148–156. 10.1016/j.prevetmed.2010.07.003 20832879 PMC3145159

