# Supplementary figures and images for: Accessory Genome Dynamics of Local and Global Staphylococcus pseudintermedius Populations
Source: Front Microbiol. 2022 Feb 10;13:798175. doi: 10.3389/fmicb.2022.798175 (PMC8867027; doi:10.3389/fmicb.2022.798175)

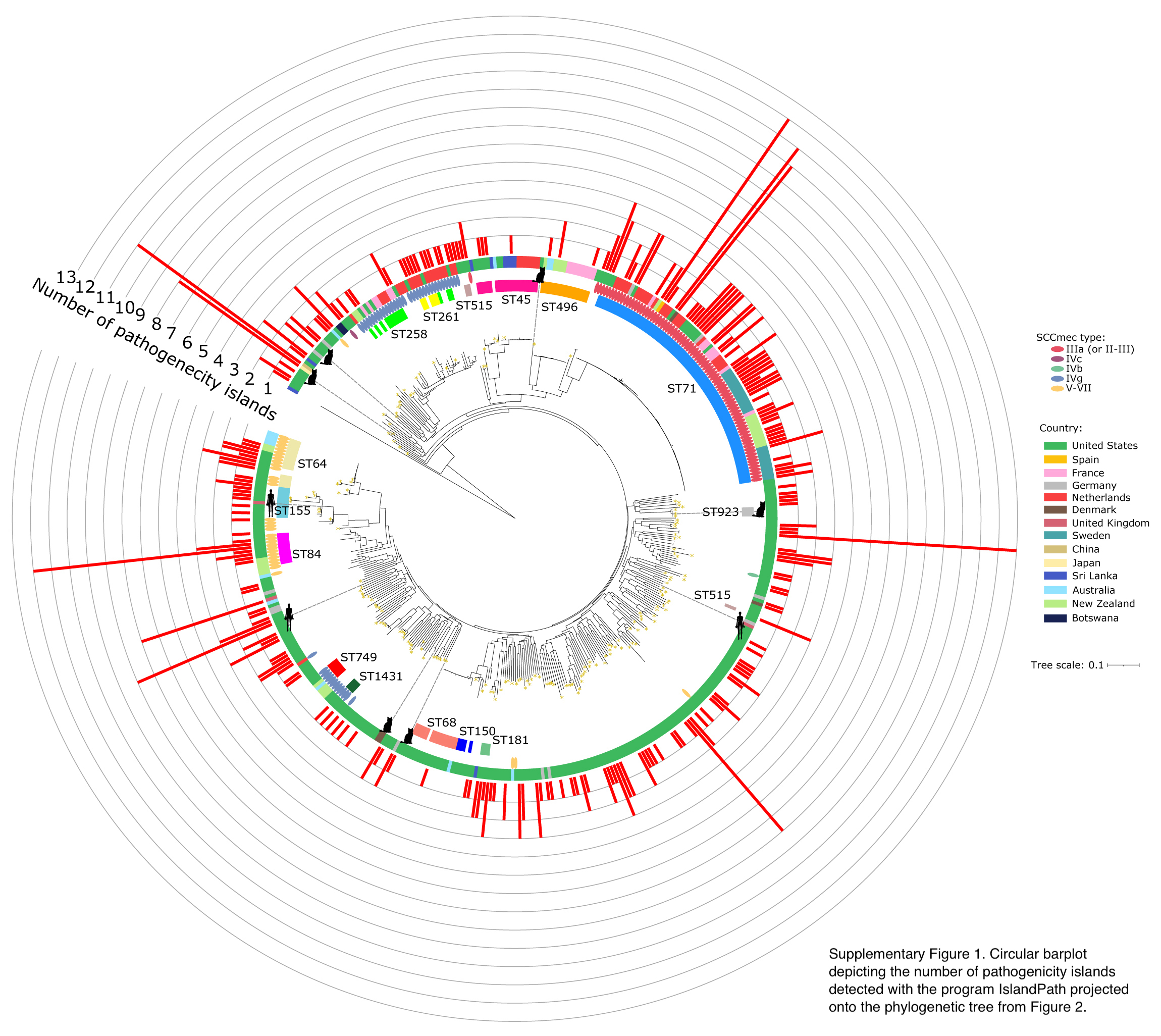

Supplement: Supplementary file 2 [file Image_1.jpeg]
